# Supplementary material for: Evaluation of flow, supply, and demand for noise reduction in urban area, Hamadan in Iran
Source: PLoS One. 2024 Jun 20;19(6):e0303581. doi: 10.1371/journal.pone.0303581 (PMC11189222; doi:10.1371/journal.pone.0303581)
Supplement: Attachment — Submitted filename: Response to Reviewers.docx [file pone.0303581.s002.docx]

**Manuscript Number:** PONE-D-23-22927

Full Title: Evaluation of flow, supply, and demand for noise reduction in urban area, Hamadan in Iran

**Dear Academic Editor, Chih-Da Wu**

we appreciate the editor and the reviewers for their detailed observations and comments on the manuscript. All changes can be seen in the manuscript. We have fulfilled their comments and suggestions and wish to submit a revised version of the manuscript for further consideration in the journal. Changes in the revised version of the manuscript are highlighted in green. Below, we also provide a point-by-point response explaining how we have addressed each of the reviewer's and editors’ comments. all tracked changes are visible in red. We hope it will be accepted. Please let me know if there are any changes required.

(supplementary data, maps, and attribute tables are ready and can be available).

Yours sincerely,

Authors

Reviewer #1:

Major Revision

Comments

1) The organization of the introduction section is not elaborate. It repeatedly mentions a single concept across various disjointed segments. Authors are required to restructure it in a more coherent manner to convey the intended message effectively. Revised
2) In the introduction, consider adding relevant statistics about the topic and study area. This can help set the context and highlight the research's significance. Because there are no statistics that completely match the topic (supply and demand), therefore, the presentation of statistics has been omitted. But it will be added if necessary.
3) The paper requires another round of proofreading to significantly enhance some sentences. A few examples are below: Grammatically, not only the few mentioned below but the entire text has been revised.

- Introduction - L31-32: One million lives and healthy years of life are lost annually in Europe due to the effect of exposure to traffic noise. Revised
- Introduction - L45-47: In general, ecosystem services are the conditions and processes through which natural ecosystems and the species that make them up, enhance human well-being. Revised
- Quantification and mapping of the demand for noise pollution reduction - L195-196: Demand analysis, based on extracting the sound level from the sound flow map and comparing it with the standard sound limits in these uses, can reflect the demand in each land use. Revised
- Discussion and Conclusion - L359-360: Therefore, sound measuring stations were selected so that the ground between the sound source and the receiver would paved and asphalted. Revised

4) In the methodology section, it would be beneficial to provide straightforward definitions for 'flow,' 'supply,' and 'demand' concerning noise reduction. Additionally, considering that the methodology contains numerous subsections, it can be challenging for readers to gain a comprehensive understanding. Therefore, it is advisable to consolidate these subsections into a single section for clarity. Done
5) In the section on quantifying and mapping traffic noise flow, it mentions the use of the Kriging method to classify noise pollution zone maps. However, there is no explanation provided for this method. Please consider adding an explanation, especially for readers who may not be familiar with it. Added
6) The quantification and mapping of the supply of noise pollution reduction section is somewhat unclear. The explanation for the two approaches is insufficient, making it difficult to follow the text. It is recommended that the authors provide a comprehensive explanation for the first approach and then proceed to explain the second one. Revised
7) In the results section, in lines 222-223, the text mentions that "The GIs were divided into six main categories: agricultural lands, gardens, parks, grass and abandoned lands, single trees, as well as street trees." To make this clearer, it would be helpful to explain the unique characteristics that define each category. Furthermore, offering an explanation for the difference between "single trees" and "street trees" would enhance understanding. Additionally, has this classification approach been used in previous studies? Revised. These 6 groups were divided based on the authors' opinion.
8) In the Results section, lines 223-224, percentages are provided for five categories (amounting to a total of 100 percent). However, what about the sixth category? Revised
9) Results contain no surprises. Have you learned anything that's not in previous, or do you just confirm common knowledge? The results obtained are seen in very few studies and no articles with this detail has investigated the effect of distance and sound barrier on noise reduction at the same time. Also, the purpose of this study was not only to investigate the effect of sound barrier and distance on noise reduction but also to introduce the method of evaluating supply and demand for this type of ecosystem service which is the first study of its kind.
10) The discussion and conclusion sections are quite lengthy and lack coherence. They tend to repeat the results from the results section. It would be beneficial to merge them with the results section to create a new section named 'Results and Discussion,' and then have a separate 'Conclusion' section encompassing the paper's summary (results and discussion), limitations, suggestions for future studies, and practical applications. Revised

Reviewer #2:

Major Revision

Comments

In my opinion, the manuscript demonstrates scientific suitability for publication. I would highly recommend conducting a thorough grammar check for the work. While the content is legible, there are still instances of poor statements that require rectification. It is advisable to include a concluding recommendation in the abstract.

Grammatically, the whole text was revised.
