# Supplementary material for: Evaluation of flow, supply, and demand for noise reduction in urban area, Hamadan in Iran
Source: PLoS One. 2024 Jun 20;19(6):e0303581. doi: 10.1371/journal.pone.0303581 (PMC11189222; doi:10.1371/journal.pone.0303581)
Supplement: Attachment — Submitted filename: Response to Reviewers'.docx [file pone.0303581.s003.docx]

**Manuscript Number:** PONE-D-23-22927

Full Title: Evaluation of flow, supply, and demand for noise reduction in urban area, Hamadan in Iran

**Dear Academic Editor, Chih-Da Wu**

we appreciate the editor and the reviewers for their detailed observations and comments on the manuscript. All changes can be seen in the manuscript. We have fulfilled their comments and suggestions and wish to submit a revised version of the manuscript for further consideration in the journal. Changes in the revised version of the manuscript are highlighted in red. Below, we also provide a point-by-point response explaining how we have addressed each reviewer's comments. We hope it will be accepted. Please let me know if there are any changes required.

(supplementary data, maps, and attribute tables are ready and can be available).

Yours sincerely,

Authors

Reviewer #1: The manuscript has shown significant improvement. However, there are still some areas that require modification. Please find my comments below:

1. Since there are numerous modifications, it is advisable to completely rewrite paragraphs instead of merely crossing out and adding new parts. In the current situation, it becomes challenging for reviewers to navigate through the manuscript. All changes have been made in previous version. The final version was attached without track changes.

2. In the introduction section, lines 75-76, the presented statistic appears inaccurate: &quot;At least 1.6 million lives are lost every year in Europe due to traffic noise exposure [1].&quot; revised

3. In line 191, could you clarify the meaning of &quot;systematically and randomly&quot; in the context of choosing the location? Revised

4. Several minor grammatical errors persist in the text. For instance.
&bull; Line 583: &quot;dstance.&quot; : Edited.

5. The title for Figure 4 requires correction. According to the legend, the traffic noise flow with barrier corresponds to the left-hand side figure. However, the title states: &quot;Sound level without considering the sound barrier effect (left).&quot; Additionally, for the right-side figure, the legend mentions &quot;traffic noise flow without barriers,&quot; while the title reads: &quot;Traffic noise flow and locations of hotspots (right).&quot; Please align the titles with the corresponding figures in the legend. Edited.
